# Structural characteristics of mitochondrial genome of *Spirobo-lus walkeri* (Spirobolida: Spirobolidae), and phylogenetic analysis of Diplopoda

**DOI:** 10.3389/fgene.2025.1566634

**Published:** 2025-03-17

**Authors:** Wenwen Zhang, Shengjun Zhao, Lingna Li, Yingzhu Li, Hongyi Liu, Peng Cui

**Affiliations:** ^1^ State Environmental Protection Scientific Observation and Research Station for Ecological Environment of Wuyi Mountains/Biodiversity Comprehensive Observation Station for Wuyi Mountains/State Environmental Protection Key Laboratory on Biosafety, Nanjing Institute of Environmental Sciences, Ministry of Ecology and Environment of China, Nanjing, China; ^2^ College of Life Sciences, Nanjing Forestry University, Nanjing, China

**Keywords:** diplopoda, mitogenome, phylogeny, *Spirobolus walkeri*, genomic features

## Abstract

The phylogeny of Diplopoda, a group of ancient arthropod and an important component of modern terrestrial ecosystems, remains unclear. Here, the complete mitogenome of *Spirobolus walkeri* was determined. The newly sequenced complete mitogenome was circular DNA molecules with sizes of 14,879 bp. The mitogenome was composed of 37 genes and one control region. Negative AT-skews and positive GC-skews were found in whole mitogenome. The gene *COX1* used CGA as the start codon, while the other PCGs utilized ATN (A, T, G) as the start codons; however, the sequence of the stop codon was variable. The Ser2 exhibited the highest usage bias. All tRNAs have typical cloverleaf structures, except *trnS-AGC* and *trnM*. Phylogenetic analysis showed that *S. walkeri* and *Spirobolus bungii* shared a close relationship and that they were also closely related with *Narceus annularus*. This study helps resolve taxonomic ambiguities among morphologically similar species and provides data to support the establishment of evolutionary benchmarks for millipedes, including gene rearrangements and variations in tRNA structure.

## 1 Introduction

Hitherto, the Diplopoda contained more than 18,000 species worldwide (Golovatch and Liu, 2019). They play the role of decomposers in ecosystems and are an important component of modern terrestrial ecosystems. Because of the large variety of species and the morphological similarity of some different species, the phylogenetic relationships among the millipede orders are uncertain despite their self-evident importance (Woo et al., 2007).

Mitochondria are fundamental organelles found in eukaryotic cells, exhibiting autonomous and preserved genetic characteristics. The mitochondrial genome (mitogenome) of most animals is circular in structure and varies in length from 13 to 17 kbp. This biomolecule typically consists of 37 coding segments (Xu et al., 2025). Specifically, it includes 13 protein-coding genes (PCGs), 22 transfer RNA genes (tRNAs), two ribosomal RNA genes (rRNAs), and one control region (Xu et al., 2024a). In recent years, mitochondrial genes as well as mitogenomes have been extensively utilized in research regarding molecular systematics, population genetics, molecular evolution, and the application of molecular barcodes in environmental investigations (Xu et al., 2024b; Xu et al., 2024c). Contrasted with mitochondrial genes, which are prone to loss, mitogenomes exhibit multiple characteristic variations that enhance the precision of inferring phylogenetic relationships. Among the features of mitogenomes, gene rearrangements are notably complex and diverse, offering significant value for phylogenetic analysis. Molecular phylogenetic methods based on mitochondrial genomes have only recently been employed to reconstruct the phylogenetic relationships of arthropods (Xu et al., 2025). Furthermore, Myriapoda species serve as excellent model organisms for exploring the relationship between gene rearrangement and phylogenetic analysis. This is due to their high frequency of mitogenome rearrangement (Woo et al., 2007). Despite their potential, studies on the mitogenomes of Diplopod species have been relatively limited. Fewer than 50 millipede species have had their mitogenomes sequenced. To conduct a comprehensive and systematic phylogenetic analysis of the class Diplopoda, a substantial number of complete mitogenomes from its subordinate species is required (Golovatch and Liu, 2019). The scarcity of mitogenome data has resulted in inadequate research into the evolutionary significance of gene rearrangement in Diplopoda and their phylogenetic relationships.

The genus *Spirobolus* is a relatively common millipede found in eastern China. Like other millipedes, they play a crucial ecological role in the formation of humus by promoting the decomposition of organic litter and maintaining soil fertility. However, few studies have focused on their evolutionary relationships, and classification methods based solely on morphology may lead to potential classification issues. Our understanding of the higher-level taxonomic relationships within the genus *Spirobolus* and their genetic composition remains limited.

*Spirobolus walkeri* (Spirobolida: Spirobolidae) was found in the eastern part of China and firstly reported and described by Pocock (PocockBassett-Smith et al., 1895). In this study, we determined the complete mitogenomes of *S. walkeri* and analyzed the mitogenomic structure with respect to reveal its phylogenetic characteristics and evolutionary relationship in Diplopoda. The purpose of this study is to provide new data for the characterization of mitotic genomes in the millipede family, to further refine the phylogenetic relationships between these groups.

## 2 Materials and methods

### 2.1 Sample collection and identification

Specimen of *S. walkeri* was collected from the Mount Wuyi in Fujian Province, China (27°34′15″N, 117°50′11″E). The sampling site was protected, and we had the permission for the sampling approved by Biodiversity Comprehensive Observation Station for Wuyi Mountains. The sample is 6 mm wide and 84 mm long, with 49 segments. The sixth and seventh segments are swollen. The first segment is covered in small indentations and striae above, laterally narrowed to 45°, with a rounded apex. The sample has a characteristic red half-ring. The specimen was identified based on the morphological characteristics described by Pocock (PocockBassett-Smith et al., 1895). Fresh tissues were dissected and stored at −20°C. The specimen was preserved in a cryogenic freezer at −80°C at the Zoology Laboratory of Nanjing Forestry University with the voucher ID H005.

### 2.2 Mitochondrial genome annotation and sequence analysis

Genomic DNA was extracted using a FastPure Cell/Tissue DNA Isolation Mini Kit (Vazyme, Nanjing, China). The complete genomic of *S. walkeri* was sequenced using the NovaSeq 6,000 sequencer on the Illumina platform (Personal, Shanghai, China) with an insert size of 300 bp (about 2 Gb of raw data). To get clean data, low-quality sequences were removed. Then, 30,878,964 reads were assembled to obtain a complete mitogenome using Geneious Prime (Version. 2021.1). The mitogenome of *Spirobolus bungii* (Accession: MT767838) was used as templates for the assembly. Sequence contigs were assembled and trimmed using the medium sensitivity/fast option in the Geneious Prime (Version. 2021.1). Consensus sequences were constructed in Geneious Prime using a 99% base call threshold. The complete mitogenome of *S. walkeri* was obtained (Accession: OR078377).

MITOS WebServer (Bernt et al., 2013) was used for sequence annotation, and all parts of the sequence were confirmed using BLAST. The RNA secondary structure was described by ViennaRNA Web Services (Kerpedjiev et al., 2015). Circular gene maps were generated using the CGView Server (Stothard et al., 2019). MEGA11 (Tamura et al., 2019) was used to determine the base composition, the relative synonymous codon usage (RSCU) of each codon, and the nonsynonymous (Ka) and synonymous substitutions (Ks) (Tamura et al., 2019). Nucleotide compositional skewness was calculated according to the formula proposed by Perna and Kocher (Ojala et al., 1981).

### 2.3 Phylogenetic analyses

The phylogenetic relationships were reconstructed using 27 Diplopoda mitogenomes with a Chilopoda mitogenome as the outgroup Bayesian inference (BI) and maximum likelihood (ML) methods were used for phylogenetic analyses with the nucleotide sequences of the 13 PCGs (Supplementary Table S3). TreeSuite was used for checking saturation. MAFFT v.7.313 (Rozewicki et al., 2019) was used to align all PCGs, and the best substitution model was identified by ModelFinder (Kalyaanamoorthy et al., 2017). The best fit models for BI and ML trees were shown in Supplementary Table S1. Phylogenetic analysis was performed using PhyloSuite v.1.2.2 (Zhang et al., 2020). The BI method was used to construct a tree using the software MrBayes (2 parallel runs, 1,000,000 generations), in which the initial 25% of sampled data were discarded as burn-in (Ronquist et al., 2012). The PSRF of the tree is close to 1 and therefore converges (Supplementary Table S2). The ML tree was constructed using IQ-TREE with 100,000 ultrafast bootstraps (Nguyen et al., 2015). The phylogenetic trees were visualized and edited using the Interactive Tree of Life Web Services (Zhou et al., 2023).

## 3 Results

### 3.1 Mitogenome structure and organization

**FIGURE 1:**
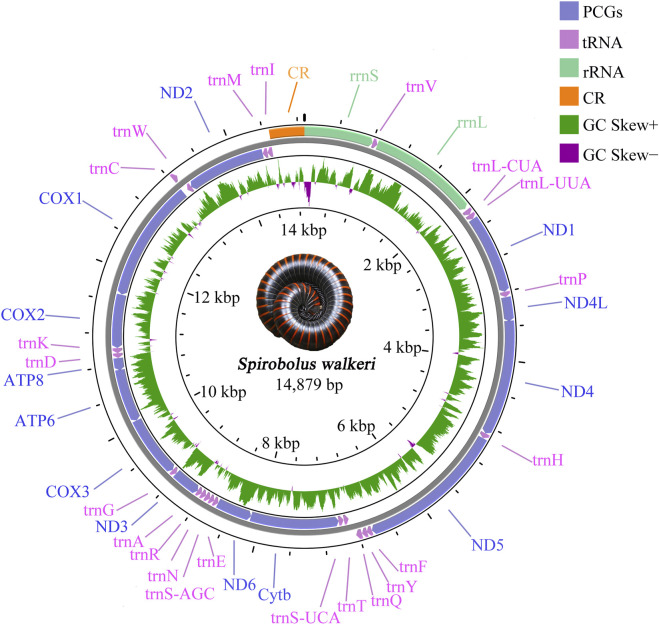
Gene map of the sequenced mitogenome of *Spirobolus walkeri*. Genes encoded by the J-chain are shown outside the circle, and those encoded by the N-chain are shown inside the circle. Different gene types are shown as filled boxes in different colours.

The complete mitogenome of *S. walkeri* (Accession: OR078377) was a double-stranded circular DNA molecule with 14,879 bp in size, which contained 37 typical mitochondrial genes (13 PCGs, 22 tRNAs, and 2 rRNAs) and one control region. Two rRNAs, 4 PCGs (*ND1, ND4L, ND4* and *ND5*), and 9 tRNAs (*trnV, trnL-CUA, trnL-UUA, trnP, trnH, trnF, trnY, trnQ,* and *trnC*) were found to be encoded on the major strand (J-chain), whereas the remaining genes were located on the minor strand (N-chain) (Figure 1). The mitogenome had 7 intervals and 11 overlaps. All intervals length ranged from 1 to 40 bp, and the longest interval occurred between *trnQ* and *trnT*. All overlaps length ranges from 1 to 8 bp, and the longest overlap occurs between *Cytb* and *ND6*, and between *trnC* and *trnW* (Supplementary Table S4).

The base composition of complete mitochondrial genome was 27.30% for A, 33.50% for T, 27.80% for G, and 11.50% for C (Supplementary Table S5). The AT-skew of *S. walkeri* was negative, while the GC-skew was positive. The AT-skew of PCGs and rRNAs showed similar features to the complete mitogenome except tRNAs. The CG-skew of complete mitogenome was negative and so is rRNAs and tRNAs, except for PCGs (Supplementary Table S6).

### 3.2 PCGs and codon usage

The total length of the 13 PCGs of *S. walkeri* was 10,988 bp. The AT-skew of the 13 PCGs was −0.128 and the GC-skew was −0.063. The base composition of the PCGs was A = 25.5%, T = 33.1%, G = 19.4%, and C = 22.0% (Supplementary Table S5). Six PCGs (*ND4L*, *ND4*, *CYTB*, *COX3*, *ATP6* and *COX2*) used ATG as the initiation codon; Three PCGs (*ND1*, *ND3* and *ND2*) used ATA as the initiation codon; and three PCGs (*ND5*, *ND6* and *ATP8*) used ATT as the initiation codon. A nonstandard initiation codon CGA was observed in *COX1* in the mitogenome of *S*. *walkeri*. Five PCGs (*ND4L*, *ND6*, *ATP8*, *COX2* and *COX1*) used TAN (TAA and TAG) as termination codon, and the other PCGs used T as the termination codon (Supplementary Table S4).

**FIGURE 2:**
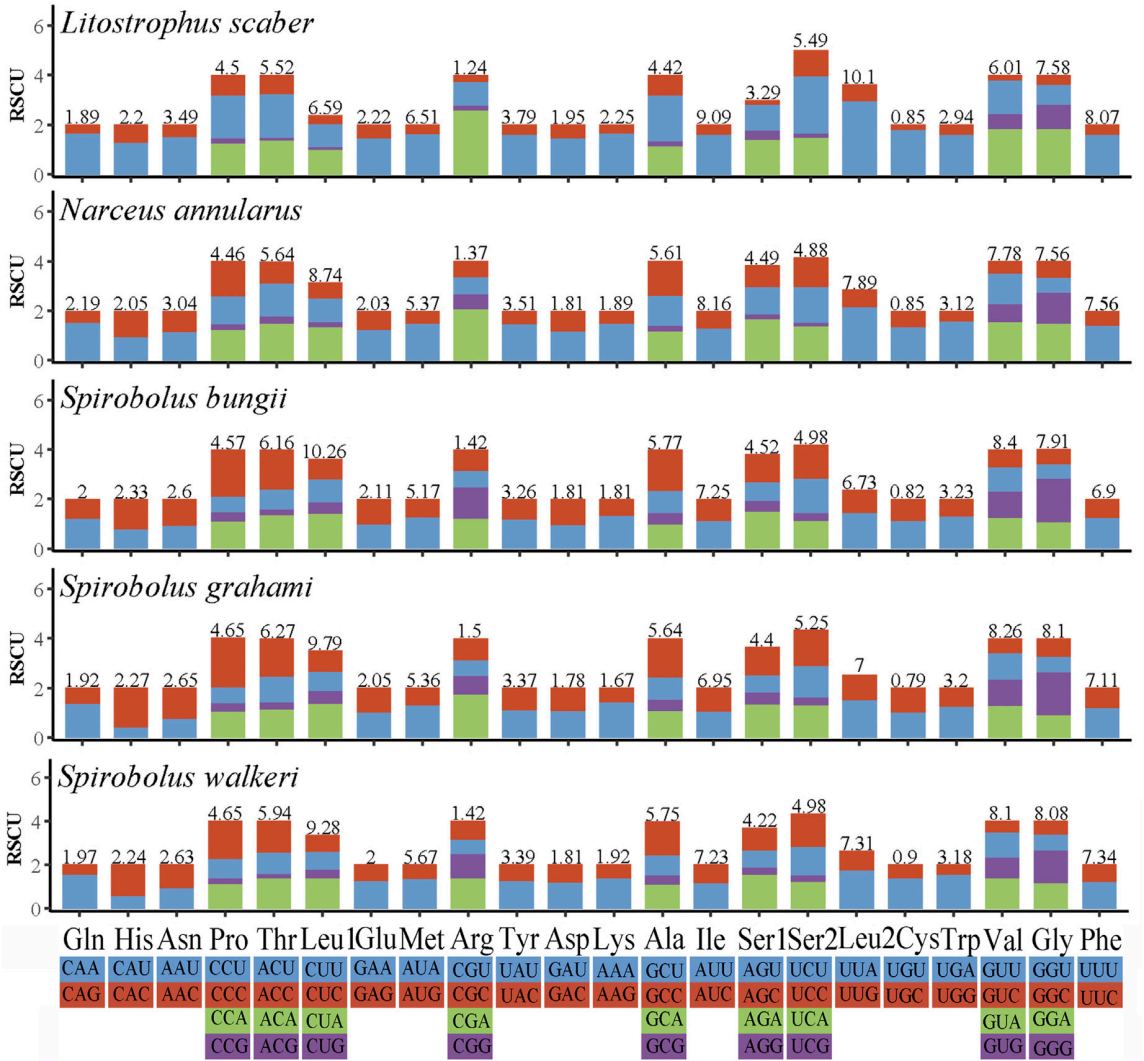
Relative synonymous codon usage in five Spirobolida mitogenomes. Different colors correspond to different third base.

The RSCU values of Spirobolida species are summarized in Figure 2. According to the analysis of RSCU and codon distribution, Leu, Val, and Gly were the three most frequently utilized amino acids, and Cys had the lowest concentration. Nine of the twenty-two amino acids had four codons, while the others had two.

**FIGURE 3:**
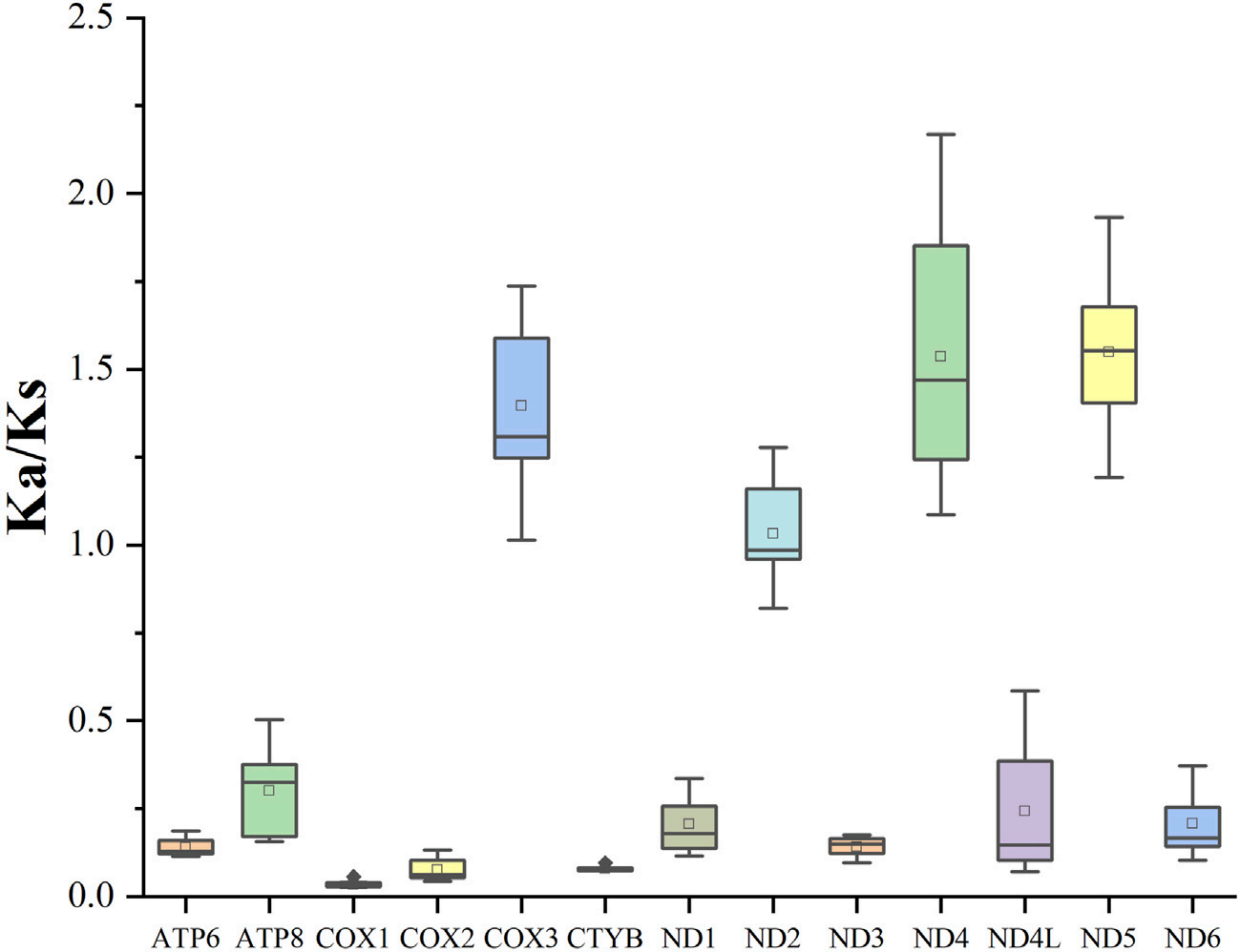
Ka/Ks values for the 13 PCGs of the order Spirobolida.

To examine the evolutionary rate of the PCGs, we evaluated the Ka/Ks values of Spirobolida species (Figure 3). The *COX3* gene (1.40), *ND4* gene (1.56), and *ND5* gene (1.55) exhibited an average Ka/Ks ratio greater than 1, while the *COX1* gene had the lowest average Ka/Ks value.

### 3.3 Transfer RNAs and ribosomal RNAs

The total size of the 22 rRNAs was 1,378 bp, with an A + T content of 66.14%, an AT-skew of 0.052, and a GC-skew of 0.075. The sizes of the 22 tRNAs ranging from 59 bp (*trnV* and *trnS-AGC*) to 68 bp (*trnQ*). All tRNAs were of the standard cloverleaf structure, except *trnM* and *trnS-AGC*. Only *trnM* showed loss of the pseudo uracil (TΨC) arm, which was lack of a simple loop (Supplementary Figure S1). Moreover, 14 U-G wobble pairs were found in tRNAs.

The length of *rrnL* was 1,270 bp, whereas *rrnS* was 802 bp. The total size of the two rRNAs was 2,072 bp, with an A + T content of 66.1%, an AT-skew of −0.063, and a GC-skew of 0.361 (Supplementary Table S6).

### 3.4 Phylogenetic analysis

**FIGURE 4:**
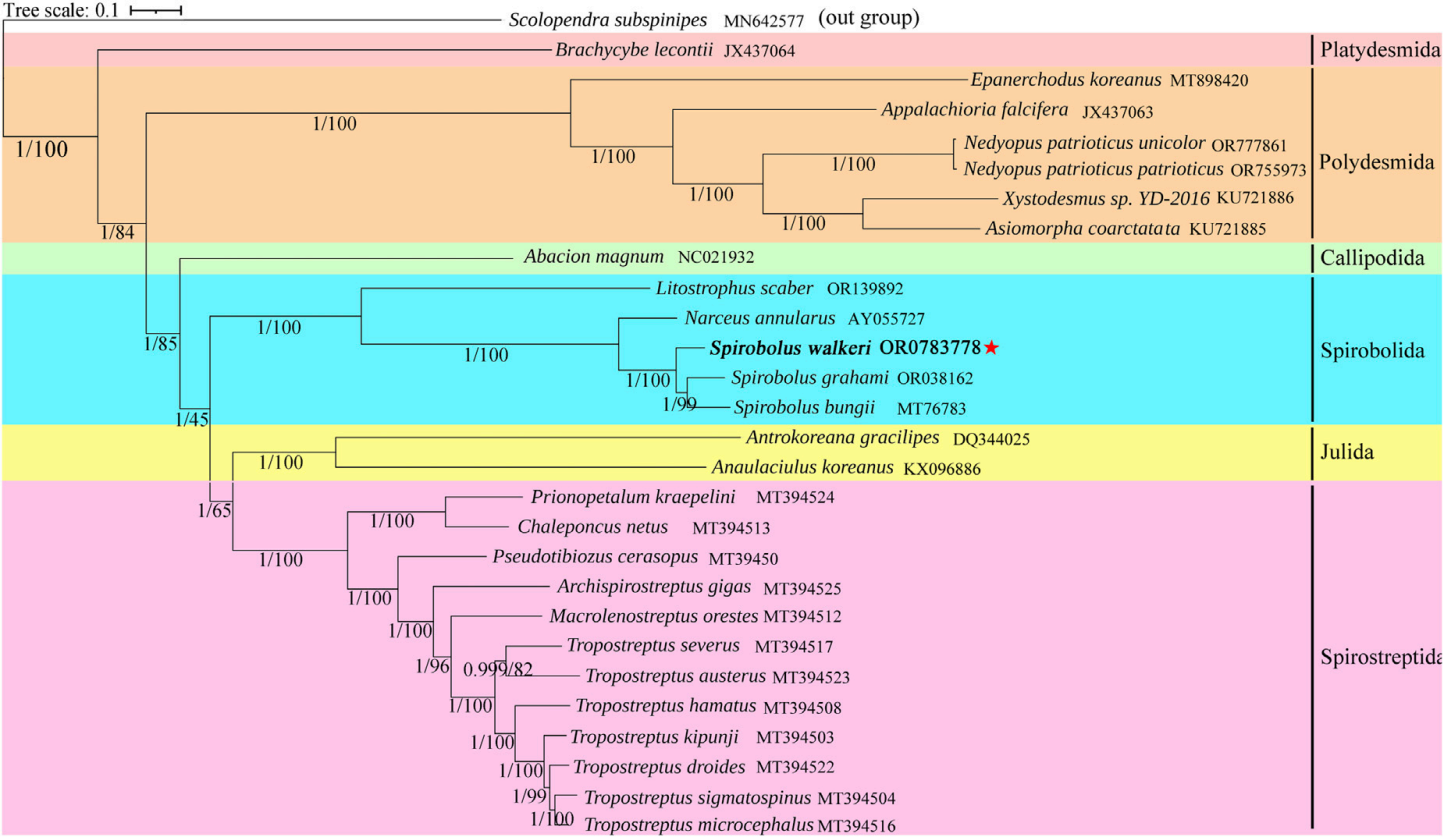
Phylogenetic tree based on 13 PCGs of 27 Diplopoda species and an outgroup. Numbers at nodes represent the posterior probability and bootstrap values for BI and ML analysis, respectively. Reported species is highlighted using bold font and red five-pointed stars.

Based on ML and BI analyses of nucleotide data of the 13 PCGs, we reconstructed the phylogenetic relationships of 27 species of Diplopoda, with *Scolopendra subspinipes* as an outgroup. As shown in Figure 4, the trees generated by BI and ML had identical topology and nodal support. *S. walkeri* clustered together with two other species in the genus, *S. bungii*, and *S. grahami*. The genus *Spirobolus* may have a sister-group relationship with the genus *Narceus*.

## 4 Discussion and conclusion

The mitogenome structure of *S. walkeri* is similar to that of other millipede mitogenomes (19). The A + T base content (61.8%) is greater than that of G + C bases (39.3%), revealing an AT preference, as observed in other Millipedes (Xu et al., 2022; Zhang WW. et al., 2024), such as *Litostrophus scaber* (Zhang et al., 2024b) and *Nedyopus patrioticus* (Zhang et al., 2024c). Additionally, the A + T% of PCGs, rRNAs, and tRNAs in Spirobolida species were also higher than the G + C%. Skewness analysis based on base composition was used to estimate the relative numbers of A to T and G to C. The results of the skewness analysis indicated that the AT-skews of Spirobolida species were negative, along with the tRNA genes, while the GC-skews were positive in relation to PCGs. This pattern is consistent with findings in other millipede species (Xu et al., 2022; Zhang WW. et al., 2024). The asymmetries in nucleotide composition are typically regarded as indicators of gene direction and replication orientation during the processes of replication and transcription (Perna and Kocher, 1995).

In the mitogenome of *S. walkeri*, PCGs are present on both the H- and L-strands. Like in *S. bungii* (Zhang WW. et al., 2024), the majority of PCGs in the *S. walkeri* mitogenome are located on the H-strand, rendering them prone to hydrolysis and oxidation. Uncommon initiation codons in *S. walkeri* have also been observed on other millipedes. For example, in *N. patrioticus*, the *ND1* gene utilizes GTA as its initiation codon (Zhang et al., 2024c). The use of T as a specific termination codon is not exclusive to one species; it is also present in other arthropods. This base can be transcribed into either TAA or TAG, serving its function during the transcription process (Ojala et al., 1981).

The RSCU values and codon distribution of *S. walkeri* were similar to species in the same order (Zhang et al., 2024b). The usage of codons ending in A or U was significantly higher than the usage of codons ending in C or G. This reflects a strong bias towards A and T nucleotides in the third position of the codons. Similarly, the preference for A and T nucleotides was reflected in the frequencies of codons. Based on the assumption of neutral protein-level evolution, the values of Ka and Ks should be equivalent, resulting in a Ka/Ks ratio of 1. When the Ka/Ks ratio is less than 1, it indicates the presence of purifying or stabilizing selection, suggesting a tendency to resist change. Conversely, a ratio greater than 1 signifies positive or Darwinian selection, which encourages evolutionary change (Hurst, 2002). Higher Ka/Ks values indicate that the genes *COX3*, *ND4*, and *ND5* are experiencing positive selection, potentially due to environmental changes or interspecific competition. Ka/Ks values close to 1 indicate that the gene *ND2* is undergoing neutral evolution, which suggests that the ND2 protein function in order Spirobolida has been highly optimized. The lowest Ka/Ks value of the *COX1* gene indicates that it has the slowest evolutionary rate, making it a useful marker for species identification (Zhang et al., 2024c).

The tRNA result is consistent with previous studies on the class Myriapoda (Ding et al., 2023; Nunes et al., 2020). All tRNAs have typical cloverleaf structures, except *trnS-AGC* and *trnM*. Moreover, *trnS-AGC* was absence of a dihydrouridine (DHU) arm, which seems to be a common phenomenon in arthropods (Bai et al., 2022; Ge et al., 2022; Jiang et al., 2022; Wu et al., 2020).

Related genes in mitogenomes are currently widely used in phylogenetic research and species classification. Mwabvu et al. (Tarombera et al., 2013) constructed a phylogenetic tree based on rrnL genes to study the evolutionary status of *Bicoxidens flavicollis*. With the widespread use of molecular biology methods, domestic and foreign researchers are increasingly studying the phylogenetics of millipedes. Nielsen et al. (2022) observed a clear genetic structure in *Tropostreptus*, with distinct lineages (both inter- and intraspecific) being defined by the mountain blocks. Moreover, phylogenetic trees provide support for the classification of the genus *Tropostreptus*. Previous research on millipede mitochondria has indicated that the genus *Tropostreptus* is phylogenetically more closely related to *Archispirostreptus gigas* and *Macrolenostreptus orestes* (Nielsen et al., 2022). Current taxonomies classify Julida, Spirostreptida, and Spirobolida as part of the superorder Juliformia; however, the precise relationships among these three groups remain controversial (Zhang et al., 2024b). A study based on mitogenome confirmed that Julida and Spirostreptida share a sister-group relationship (Zuo et al., 2022). However, morphological studies suggest that Spirobolida and Julida have a sister-group relationship (Blanke and Wesener, 2014). Our results align with previous phylogenetic studies based on mitogenome. Although mitogenome exhibited several distinctive variations that improved the accuracy of inferring phylogenetic relationships, our results were relatively weak, possibly due to limited use of mitogenome data. More mitogenomes would significantly enhance phylogenetic studies of Diplopoda species.

In general, the study of *S. walkeri*’s mitogenome elucidate its evolutionary dynamics through gene arrangement, selection pressures, and nucleotide diversity, thereby revealing its divergence within the *Spirobolus* genus and its environmental adaptations. The complete mitogenome of *S. walkeri* will benefit resolving taxonomic ambiguities among morphologically similar species and provides data to support the establishment of evolutionary benchmarks for millipedes, including gene rearrangements and variations in tRNA structure. This research addresses gaps in genetic resources and provides a significant molecular foundation for soil invertebrate conservation, biogeography, and studies on global change.

## Data Availability

The datasets presented in this study can be found in online repositories. The names of the repository/repositories and accession number(s) can be found below: https://www.ncbi.nlm.nih.gov/genbank/, OR078377.
