# Supplementary material for: Structural characteristics of mitochondrial genome of Spirobo-lus walkeri (Spirobolida: Spirobolidae), and phylogenetic analysis of Diplopoda
Source: Front Genet. 2025 Mar 17;16:1566634. doi: 10.3389/fgene.2025.1566634 (PMC11955670; doi:10.3389/fgene.2025.1566634)
Supplement: Supplementary file 1 [file Supplementaryfile1.docx]

Supplementary Material

# Supplementary Figures and Tables

## Supplementary Figures

**
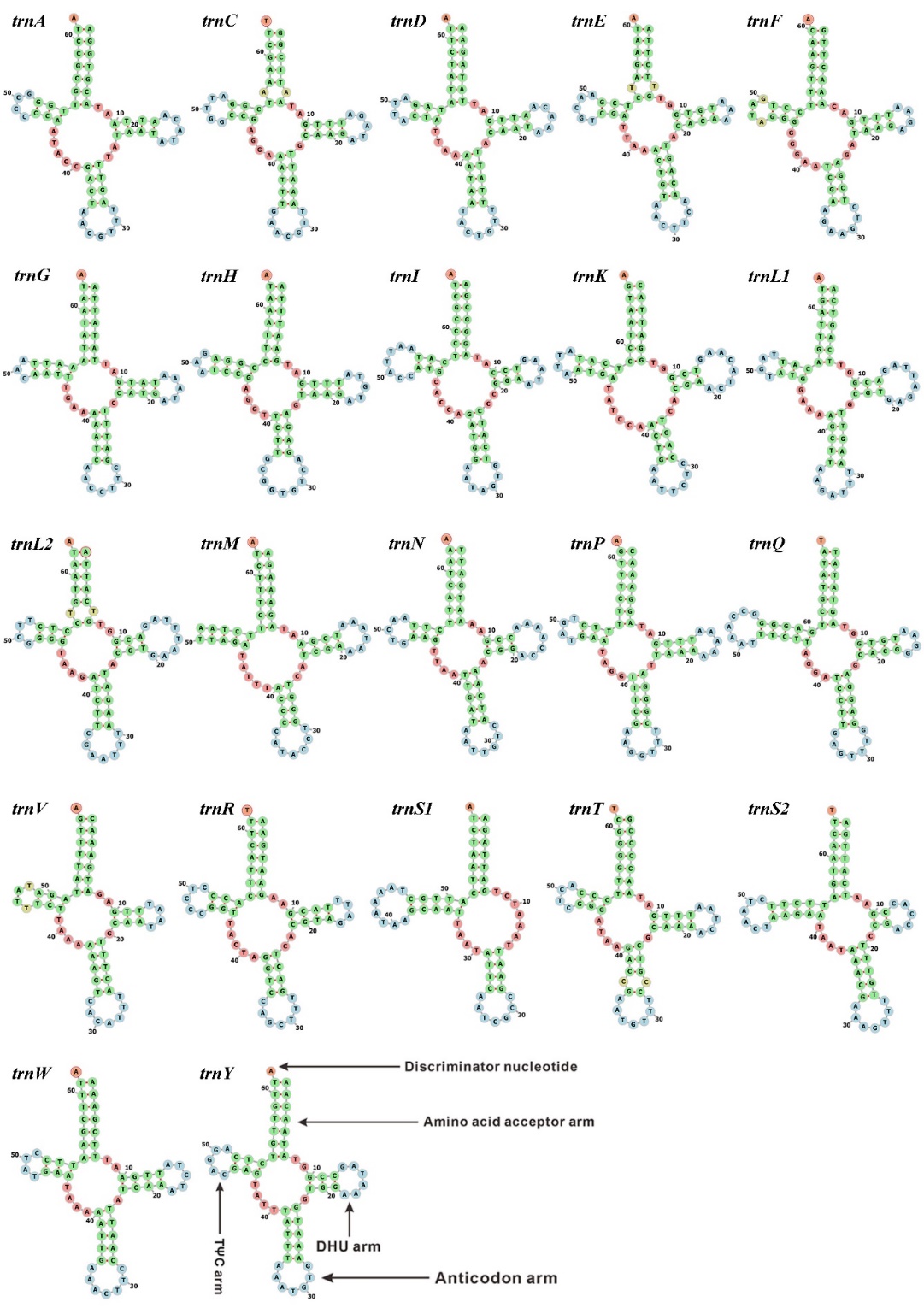
**

**Figure S1.** Secondary structures of the 22 transfer RNA genes of *Spirobolus walkeri*.

## Supplementary Tables

| Tree | Subset partitions | Best-fit model |
| --- | --- | --- |
| ML | CYTB, ATP6, COX2, trnT, trnS2, trnQ, trnN, trnM, trnL, trnH, trnG, trnF, trnD | TIM2+F+I+G4 |
|  | NAD1, ND4, ND5 | GTR+F+R5 |
|  | ND4L, ND6, ND2, ND3 | TPM3u+F+I+G4 |
|  | ATP8, trnS1 | TPM2u+F+G4 |
|  | COX1, COX3, trnS, trnL2, trnK, trnE, trnA | TIM2+F+R5 |
|  | trnY, trnW, trnV, trnR, trnP, trnL1, trnI, trnC, rrnS, rrnL | GTR+F+I+I+R4 |
| BI | CYTB, ATP6, COX2, trnT, trnS2, trnQ, trnN, trnM, trnL, trnH, trnG, trnF, trnD | GTR+F+I+G4 |
|  | ND1, ND4, ND5 | GTR+F+I+G4 |
|  | ND4L, ND6, ND2, ND3 | GTR+F+I+G4 |
|  | ATP8, trnS1 | HKY+F+G4 |
|  | COX1, COX3, trnS2, trnL2, trnK, trnE, trnA, | GTR+F+I+G4 |
|  | trnY, trnW, trnV, trnR, trnP, trnL1, trnI, trnC, rrnS, rrnL | GTR+F+I+G4 |

**Table S1**: The best-fit models obtained using ModelFinder for ML and BI trees.

**Table S2.** Summary statistics for branch and node parameters

| Parameter | Mean | Variance | Lower | Upper | Median | PSRF+ | Nruns |
| --- | --- | --- | --- | --- | --- | --- | --- |
| length{1}[1] | 0.190385 | 0.09991 | 0.000021 | 0.770748 | 0.078763 | 1 | 2 |
| length{1}[2] | 0.194552 | 0.113617 | 0.000025 | 0.770566 | 0.078253 | 1 | 2 |
| length{1}[3] | 0.189022 | 0.123269 | 0.000015 | 0.755135 | 0.068626 | 1 | 2 |
| length{1}[4] | 0.178223 | 0.087771 | 0.000017 | 0.703977 | 0.074107 | 1 | 2 |
| length{1}[5] | 0.188488 | 0.117409 | 0.000001 | 0.749155 | 0.072857 | 1.001 | 2 |
| length{1}[6] | 0.189523 | 0.103044 | 0 | 0.743808 | 0.075101 | 1 | 2 |
| length{1}[7] | 0.191591 | 0.113423 | 0.000001 | 0.749668 | 0.075682 | 1.001 | 2 |
| length{1}[8] | 0.197531 | 0.133399 | 0.000008 | 0.766042 | 0.076075 | 1.001 | 2 |
| length{1}[9] | 0.181125 | 0.086506 | 0.000008 | 0.729758 | 0.07089 | 1 | 2 |
| length{1}[10] | 0.192589 | 0.109322 | 0.000001 | 0.768898 | 0.075377 | 1.003 | 2 |
| length{1}[11] | 0.177891 | 0.099769 | 0.000017 | 0.731009 | 0.070417 | 1.003 | 2 |
| length{1}[12] | 0.179256 | 0.097054 | 0.000009 | 0.705455 | 0.076294 | 1.001 | 2 |
| length{1}[13] | 0.178258 | 0.09785 | 0.000003 | 0.704739 | 0.068665 | 1.001 | 2 |
| length{1}[14] | 0.183213 | 0.09799 | 0.000001 | 0.715453 | 0.076259 | 1.002 | 2 |
| length{1}[15] | 0.178596 | 0.088335 | 0.00001 | 0.707724 | 0.073923 | 1.004 | 2 |
| length{1}[16] | 0.191161 | 0.104943 | 0.000012 | 0.761155 | 0.073446 | 1.001 | 2 |
| length{1}[17] | 0.185169 | 0.098156 | 0.000005 | 0.773018 | 0.074853 | 1 | 2 |
| length{1}[18] | 0.191683 | 0.137999 | 0.000005 | 0.730955 | 0.07704 | 1 | 2 |
| length{1}[19] | 0.192992 | 0.101442 | 0.000019 | 0.755673 | 0.079568 | 1 | 2 |
| length{1}[20] | 0.179321 | 0.094898 | 0.000022 | 0.707843 | 0.071559 | 1 | 2 |
| length{1}[21] | 0.181619 | 0.116472 | 0.000007 | 0.740185 | 0.069212 | 1.001 | 2 |
| length{1}[22] | 0.192392 | 0.120748 | 0.000014 | 0.763814 | 0.077348 | 1.001 | 2 |
| length{1}[23] | 0.187489 | 0.097549 | 0.000026 | 0.732487 | 0.07654 | 1 | 2 |
| length{1}[24] | 0.191978 | 0.120752 | 0.000009 | 0.821717 | 0.07061 | 1 | 2 |
| length{1}[25] | 0.188637 | 0.103605 | 0.000021 | 0.71 | 0.074002 | 1 | 2 |
| length{1}[26] | 0.185055 | 0.109862 | 0.000011 | 0.719677 | 0.072221 | 1.004 | 2 |
| length{1}[27] | 0.180119 | 0.087902 | 0.000001 | 0.721425 | 0.075068 | 1 | 2 |
| length{1}[28] | 0.177513 | 0.091973 | 0.000005 | 0.685867 | 0.06958 | 1 | 2 |
| length{1}[29] | 0.189053 | 0.100327 | 0.000009 | 0.763866 | 0.075383 | 1 | 2 |
| length{1}[30] | 0.183035 | 0.112376 | 0.000001 | 0.756861 | 0.07054 | 1 | 2 |
| length{1}[31] | 0.187141 | 0.101632 | 0.000004 | 0.76357 | 0.074407 | 1.003 | 2 |
| length{1}[32] | 0.188458 | 0.108656 | 0.000013 | 0.776071 | 0.073664 | 1 | 2 |
| length{1}[33] | 0.186086 | 0.099943 | 0.000008 | 0.779108 | 0.069676 | 1 | 2 |
| length{1}[34] | 0.189168 | 0.115678 | 0.000008 | 0.750676 | 0.077024 | 1 | 2 |
| length{1}[35] | 0.180289 | 0.082274 | 0.000005 | 0.715981 | 0.075596 | 1.002 | 2 |
| length{1}[36] | 0.18761 | 0.111148 | 0.000021 | 0.779202 | 0.072609 | 1 | 2 |
| length{1}[37] | 0.184694 | 0.090106 | 0.000022 | 0.752921 | 0.073949 | 1 | 2 |
| length{1}[38] | 0.186938 | 0.087525 | 0.000003 | 0.74002 | 0.075901 | 1.002 | 2 |
| length{1}[39] | 0.188331 | 0.109076 | 0.000007 | 0.744033 | 0.076544 | 1 | 2 |
| length{1}[40] | 0.185802 | 0.091583 | 0.000013 | 0.755027 | 0.077281 | 1 | 2 |
| length{1}[41] | 0.193546 | 0.118945 | 0.000006 | 0.800651 | 0.076339 | 1.002 | 2 |
| length{1}[42] | 0.176522 | 0.070246 | 0.000002 | 0.666396 | 0.076455 | 1.002 | 2 |
| length{1}[43] | 0.183472 | 0.091213 | 0.000002 | 0.730392 | 0.075478 | 1.001 | 2 |
| length{1}[44] | 0.179928 | 0.090518 | 0.000004 | 0.723802 | 0.074292 | 1.001 | 2 |
| length{1}[45] | 0.185131 | 0.097828 | 0.000006 | 0.726657 | 0.071224 | 1 | 2 |
| length{1}[46] | 0.17588 | 0.080126 | 0.000015 | 0.692517 | 0.074083 | 1 | 2 |
| length{1}[47] | 0.191831 | 0.121226 | 0.000005 | 0.77316 | 0.072553 | 1 | 2 |
| length{1}[48] | 0.176325 | 0.08164 | 0.000004 | 0.658432 | 0.076795 | 1.001 | 2 |
| length{1}[49] | 0.182751 | 0.086873 | 0.000016 | 0.732193 | 0.07681 | 1 | 2 |
| length{1}[50] | 0.180948 | 0.086651 | 0.00002 | 0.699089 | 0.072143 | 1.001 | 2 |
| length{1}[51] | 0.190835 | 0.109161 | 0.00001 | 0.738294 | 0.073115 | 1 | 2 |
| length{1}[52] | 0.184743 | 0.091127 | 0.000002 | 0.716539 | 0.076821 | 1 | 2 |
| length{1}[53] | 0.190167 | 0.09794 | 0.000002 | 0.774817 | 0.07503 | 1 | 2 |
| length{1}[54] | 0.183402 | 0.097396 | 0.000011 | 0.713807 | 0.076357 | 1 | 2 |
| length{1}[55] | 0.181009 | 0.088229 | 0.000001 | 0.714795 | 0.074697 | 1.002 | 2 |

**Table S3.** List of the mitogenomes analyzed in this study.

| Class | Order | Species | Accession no. |
| --- | --- | --- | --- |
| Diplopoda | Spirostreptida | *Archispirostreptus gigas* | MT394525 |
|  |  | *Macrolenostreptus orestes* | MT394512 |
|  |  | *Prionopetalum kraepelini* | MT394524 |
|  |  | *Pseudotibiozus cerasopus* | MT394506 |
|  |  | *Tropostreptus austerus* | MT394523 |
|  |  | *Tropostreptus droides* | MT394522 |
|  |  | *Tropostreptus hamatus* | MT394508 |
|  |  | *Tropostreptus kipunji* | MT394503 |
|  |  | *Tropostreptus microcephalus* | MT394516 |
|  |  | *Tropostreptus severus* | MT394517 |
|  |  | *Tropostreptus sigmatospinus* | MT394504 |
|  |  | *Chaleponcus netus* | MT394513 |
|  | Spirobolida | *Narceus annularus* | AY055727 |
|  |  | *Litostrophus scaber* | OR139892 |
|  |  | *Spirobolus grahami* | OR038162 |
|  |  | *Spirobolus bungii* | MT767838 |
|  |  | *Spirobolus walkeri* | OR078377 |
|  | Callipodida | *Abacion magnum* | NC021932 |
|  | Polydesmida | *Asiomorpha coarctata* | KU721885 |
|  |  | *Epanerchodus koreanus* | MT898420 |
|  |  | *Appalachioria falcifera* | JX437063 |
|  |  | *Nedyous patrioticus unicolor* | OR777861 |
|  |  | *Nedyous patrioticus patrioticus* | OR755973 |
|  |  | *Xystodesmus* sp. YD-2016 | KU721886 |
|  | Julida | *Anaulaciulus koreanus* | KX096886 |
|  |  | *Antrokoreana gracilipes* | DQ344025 |
|  | Platydesmida | *Brachycybe lecontii* | JX437064 |
| Chilopoda |  | *Scolopendra subspinipes* | MN642577 |

| Gene | Location | | Intergenic nucleotides | Size | Codon | | Stand |
| --- | --- | --- | --- | --- | --- | --- | --- |
|  | From | To |  |  | Start | Stop |  |
| rrnS | 1/12/1/1 | 802/814/757/784 | -/11/-/- | 802/803/757/784 |  |  | +/+/+/+ |
| trnV | 803/815/757/785 | 861/873/875/843 | 0/0/0/0 | 59/59/119/59 |  |  | +/+/+/+ |
| rrnL | 862/874/979/844 | 2131/2143/2011/2134 | 0/0/104/0 | 1270/1270/1033/1291 |  |  | +/+/+/+ |
| trnL-CUA | 2153/2165/2103/2135 | 2215/2227/2165/2197 | 21/21/92/0 | 63/63/63/63 |  |  | +/+/+/+ |
| trnL-UUA | 2216/2228/2166/2198 | 2278/2290/2228/2260 | 0/0/1/0 | 63/63/63/63 |  |  | +/+/+/+ |
| ND1 | 2279/2291/2229/2261 | 3200/3212/3150/3182 | 0/0/1/0 | 922/922/922/922 | ATA/ATA/ATA/ATA | T/T/T/T | +/+/+/+ |
| trnP | 3201/3213/3151/3183 | 3262/3275/3213/3244 | 0/0/1/0 | 62/63/63/62 |  |  | +/+/+/+ |
| ND4L | 3264/3277/3215/3246 | 3545/3558/3496/3527 | 1/1/2/1 | 282/282/282/282 | ATG/ATG/ATG/ATG | TAG/TAG/TAG/TAG | +/+/+/+ |
| ND4 | 3539/3552/3490/3521 | 4883/4893/4834/4859 | -7/-7/-6/-7 | 1345/1342/1345/1339 | ATG/ATG/ATG/ATG | T/T/T/T | +/+/+/+ |
| trnH | 4884/4894/4835/4860 | 4946/4956/4897/4921 | 0/0/1/0 | 63/63/63/62 |  |  | +/+/+/+ |
| ND5 | 4947/4957/4898/4922 | 6648/6658/6599/6623 | 0/0/1/0 | 1702/1702/1702/1702 | ATT/ATT/ATT/ATT | T/TAA/T/T | +/+/+/+ |
| trnF | 6649/6659/6600/6624 | 6709/6719/6660/6684 | 0/0/1/0 | 61/61/61/61 |  |  | +/+/+/+ |
| trnY | 6706/6716/6657/6681 | 6767/6777/6718/6742 | -4/-4/-3/-4 | 62/62/62/62 |  |  | +/+/+/+ |
| trnQ | 6770/6780/6721/6745 | 6837/6847/6788/6811 | 2/2/3/2 | 68/68/68/67 |  |  | +/+/+/+ |
| trnT | 6878/6888/6829/6851 | 6939/6948/6890/6912 | 40/40/41/39 | 62/61/62/62 |  |  | -/-/-/- |
| trnS-UCA | 6944/6953/6895/6917 | 7007/7016/6958/6980 | 4/4/5/4 | 64/64/64/64 |  |  | -/-/-/- |
| Cytb | 7008/7017/6959/6979 | 8124/8133/8075/8097 | 0/0/1/-2 | 1117/1117/1117/1119 | ATG/ATG/ATG/ATG | T/T/T/TAG | -/-/-/- |
| ND6 | 8117/8126/8068/8098 | 8572/8581/8523/8545 | -8/-8/-7/0 | 456/456/456/448 | ATT/ATT/ATT/ATT/ATT | TAA/TAA/TAA/T/TAA | -/-/-/- |
| trnE | 8573/8582/8524/8546 | 8633/8642/8584/8606 | 0/0/1/0/0 | 61/61/61/61 |  |  | -/-/-/- |
| trnS-AGC | 8633/8643/8585/8607 | 8691/8699/8641/8663 | -1/0/1/0 | 59/57/57/57 |  |  | -/-/-/- |
| trnN | 8691/8700/8642/8664 | 8753/8762/8704/8726 | -1/0/1/0 | 63/63/63/63 |  |  | -/-/-/- |
| trnR | 8753/8762/8704/8726 | 8814/8823/8765/8786 | -1/-1/0/-1 | 62/62/62/61 |  |  | -/-/-/- |
| trnA | 8814/8823/8765/8786 | 8875/8884/8826/8846 | -1/-1/0/-1 | 62/62/62/61 |  |  | -/-/-/- |
| ND3 | 8876/8885/8827/8846 | 9221/9230/9172/9193 | 0/0/1/-1 | 346/346/346/348 | ATA/ATT/ATA/ATA | T/T/T/T | -/-/-/- |
| trnG | 9222/9231/9173/9194 | 9284/9293/9235/9256 | 0/0/1/0 | 63/63/63/63 |  |  | -/-/-/- |
| COX3 | 9285/9294/9236/9257 | 10,062/10,071/10,013/10,034 | 0/0/1/0 | 778/778/778/778 | ATG/ATG/ATG/ATG | T/T/T/T | -/-/-/- |
| ATP6 | 10,063/10,072/10,014/10,035 | 10,738/10,747/10,689/10,710 | 0/0/1/0 | 676/676/676/676 | ATG/ATG/ATG/ATG | T/T/T/T | -/-/-/- |
| ATP8 | 10,732/10,741/10,683/10,704 | 10,88710,896/10,838/10,859 | -7/-7/-6/-7 | 156/156/156/156 | ATT/ATT/ATT/ATT | TAA/TAA/TAA/TAA | -/-/-/- |
| trnD | 10,888/10,987/10,839/10,860 | 10,949/10,958/10,900/10,921 | 0/0/1/0 | 62/62/62/62 |  |  | -/-/-/- |
| trnK | 10,949/10,958/10,900/10,921 | 11,014/11,023/10,965/10,984 | -1/-1/0/-1 | 66/66/66/64 |  |  | -/-/-/- |
| COX2 | 11,015/11,024/10,966/10,985 | 11,692/11,701/11,643/11,662 | 0/0/1/0 | 678/678/678/678 | ATG/ATG/ATG/ATG | TAA/TAA/TAA/TAA | -/-/-/- |
| COX1 | 11,696/11,705/11,647/11,666 | 13,225/13,234/13,176/13,198 | 3/3/4/3 | 1530/1530/1530/1533 | CGA/CGA/CGA/ACG | TAA/TAA/TAA/TAA | -/-/-/- |
| trnC | 13,231/13,240/13,182/13,201 | 13,293/13,302/13,244/13,263 | 5/5/6/2 | 63/63/63/63 |  |  | +/+/+/+ |
| trnW | 13,286/13,295/13,237/13,256 | 13,347/13,356/13,298/13,317 | -8/-8/-7/-8 | 62/62/62/62 |  |  | -/-/-/- |
| ND2 | 13,348/13,357/13,299/13,316 | 14,347/14,356/14,298/14,314 | 0/0/1/-2 | 1000/1000/1000/999 | ATA/ATA/ATA/ATA | T/T/T/TAA | -/-/-/- |
| trnM | 14,348/14,357/14,299/14,315 | 14,410/14,419/14,361/14,377 | 0/0/1/0 | 63/63/63/63 |  |  | -/-/-/- |
| trnI | 14,410/14,420/14,362/14,377 | 14,474/14,483/14,425/14,441 | -1/0/1/-1 | 65/64/64/65 |  |  | -/-/-/- |
| CR | 14,475/14,484/14,426/14,442 | 14,879/14,879/14,875/14,868 | 0/0/1/0 | 405/395/450/427 |  |  |  |

**Table S4** Features of the mitogenomes of *S. walkeri*, *S. bungii*, *S. graham*, and *N.annularus*.

| Region | A% | T% | AT-Skew | G% | C% | GC-Skew |
| --- | --- | --- | --- | --- | --- | --- |
| Whole mitogenome | 27.3 | 33.5 | -0.102 | 27.8 | 11.5 | 0.415 |
| PCGs | 25.5 | 33.1 | -0.130 | 19.4 | 22.0 | -0.063 |
| rRNAs | 31 | 35.1 | -0.062 | 22.8 | 11.1 | 0.345 |
| tRNAs | 34.8 | 31.3 | 0.053 | 18.2 | 15.7 | 0.074 |

**Table S5.** Base composition of the complete genomes, protein-coding genes (PCGs), rRNAs and tRNAs of *Spirobolus walkeri* mitogenome.

**Table S6**. Base composition of the complete genomes, protein-coding genes (PCGs), rRNAs and tRNAs of the five Spirobolida mitogenomes.

| **Region** | **Species** | **Length (bp)** | **A+T%** | **AT-Skew** | **GC-Skew** |
| --- | --- | --- | --- | --- | --- |
| Whole mitogenome | *Litostrophus scaber* | 15,081 | 69.31 | -0.016 | 0.414 |
|  | *Narceus annularus* | 14,868 | 63.81 | 0.069 | -0.397 |
|  | *Spirobolus bungii* | 14,879 | 59.21 | -0.102 | 0.395 |
|  | *Spirobolus grahami* | 14,875 | 58.73 | -0.113 | 0.415 |
|  | *Spirobolus walkeri* | 14,879 | 60.82 | -0.102 | 0.417 |
| PCGs | *Litostrophus scaber* | 10,947 | 68.01 | -0.153 | -0.017 |
|  | *Narceus annularus* | 10,974 | 62.12 | -0.132 | -0.059 |
|  | *Spirobolus bungii* | 10,977 | 56.71 | -0.135 | -0.054 |
|  | *Spirobolus grahami* | 10,989 | 56.51 | -0.136 | -0.063 |
|  | *Spirobolus walkeri* | 10,980 | 58. 76 | -0.128 | -0.063 |
| rRNAs | *Litostrophus scaber* | 1788 | 71.5 | -0.046 | 0.413 |
|  | *Narceus annularus* | 2,075 | 68.7 | -0.065 | 0.374 |
|  | *Spirobolus bungii* | 2,073 | 66.12 | -0.062 | 0.345 |
|  | *Spirobolus grahami* | 1,790 | 63.84 | -0.085 | 0.359 |
|  | *Spirobolus walkeri* | 2,072 | 66.02 | -0.063 | 0.361 |
| tRNAs | *Litostrophus scaber* | 1,388 | 73.1 | 0.031 | 0.078 |
|  | *Narceus annularus* | 1,370 | 67.1 | 0.035 | 0.098 |
|  | *Spirobolus bungii* | 1,375 | 65.05 | 0.056 | 0.089 |
|  | *Spirobolus grahami* | 1,376 | 64.82 | 0.055 | 0.061 |
|  | *Spirobolus walkeri* | 1,378 | 66.14 | 0.052 | 0.075 |
